# Pneumocephalus: a rare and life-threatening, but reversible, complication after penetrating lumbar injury

**DOI:** 10.1007/s00701-018-03796-y

**Published:** 2019-01-17

**Authors:** Zora Gorissen, Karlijn Hakvoort, Mark van den Boogaart, Sylvia Klinkenberg, Olaf Schijns

**Affiliations:** 1 Faculty of Health, Medicine & Life Sciences (FHML), Maastricht University, Maastricht, The Netherlands; 2 Department of Neurosurgery, Uniklinik RWTH Aachen, Klinik für Neurochirurgie, Pauwelsstraße 20, 52074 Aachen, Germany; 3 Department of Orthopaedic Surgery, Maastricht University Medical Center, PO Box 5800, 6202 AZ Maastricht, The Netherlands; 4 Department of Neurology, Maastricht University Medical Center, PO Box 5800, 6202 AZ Maastricht, The Netherlands; 5 Department of Neurosurgery, Maastricht University Medical Center, PO Box 5800, 6202 AZ Maastricht, The Netherlands; 6 School for Mental Health and Neuroscience (MHeNs), Maastricht University, Universiteitssingel 40, 6229 ER Maastricht, The Netherlands

**Keywords:** Pneumocephalus, Tension pneumocephalus, Spinal trauma, Pediatric patients

## Abstract

Pneumocephalus, the presence of intracranial air, is a complication especially seen after neurotrauma or brain surgery. When it leads to a pressure gradient, a so-called tension pneumocephalus, it may require emergency surgery. Clinical symptomatology, especially in young children, does not differentiate between a pneumocephalus and a tension pneumocephalus. An additional CT scan is therefore warranted. Here, we report on a rare case of pneumocephalus after penetrating lumbar injury. Additionally, the pathophysiology of pneumocephalus, as well as its recommendations for diagnosis and treatment, will be elucidated.

## Case

A 6-year-old boy without relevant medical history presented at the emergency department of the Maastricht University Medical Centre (MUMC+), after referral from a local hospital. Several hours before, the boy fell off a 1-m-high windowsill in his house and landed with his back on a protrusion of the central heating. In the local hospital emergency room, he complained of a painful and continuously leaking wound on his back. At that moment, the boy showed no signs of impaired consciousness or any neurological deficit. After transfer, at presentation in the MUMC+, he was drowsy with a varying decreased Glasgow Coma Score of 10 (E2M6V2) to 13. Furthermore, he presented with episodes of bradycardia and a preferential head position towards the left. Motor and sensory functions were undisturbed, and deep tendon reflexes were symmetrical and normal, with no Babinski signs.

**Fig. 1:**
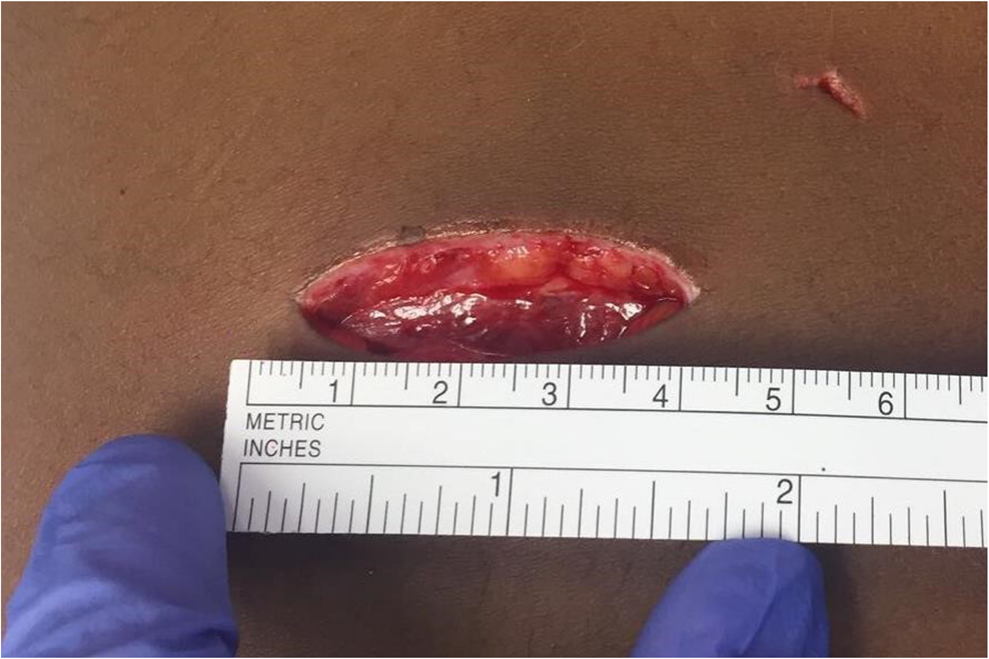
Horizontally oriented lumbar wound

Physical examination showed a horizontally oriented, deep, and sharp confined wound of about 4 cm in length, located paravertebrally at the lower lumbar region (Fig. 1). Due to penetration of the subcutis, fascia, and paravertebral muscles, the spinous process was visible and the wound was continuously leaking bloody fluid.

## Imaging

**Fig. 2:**
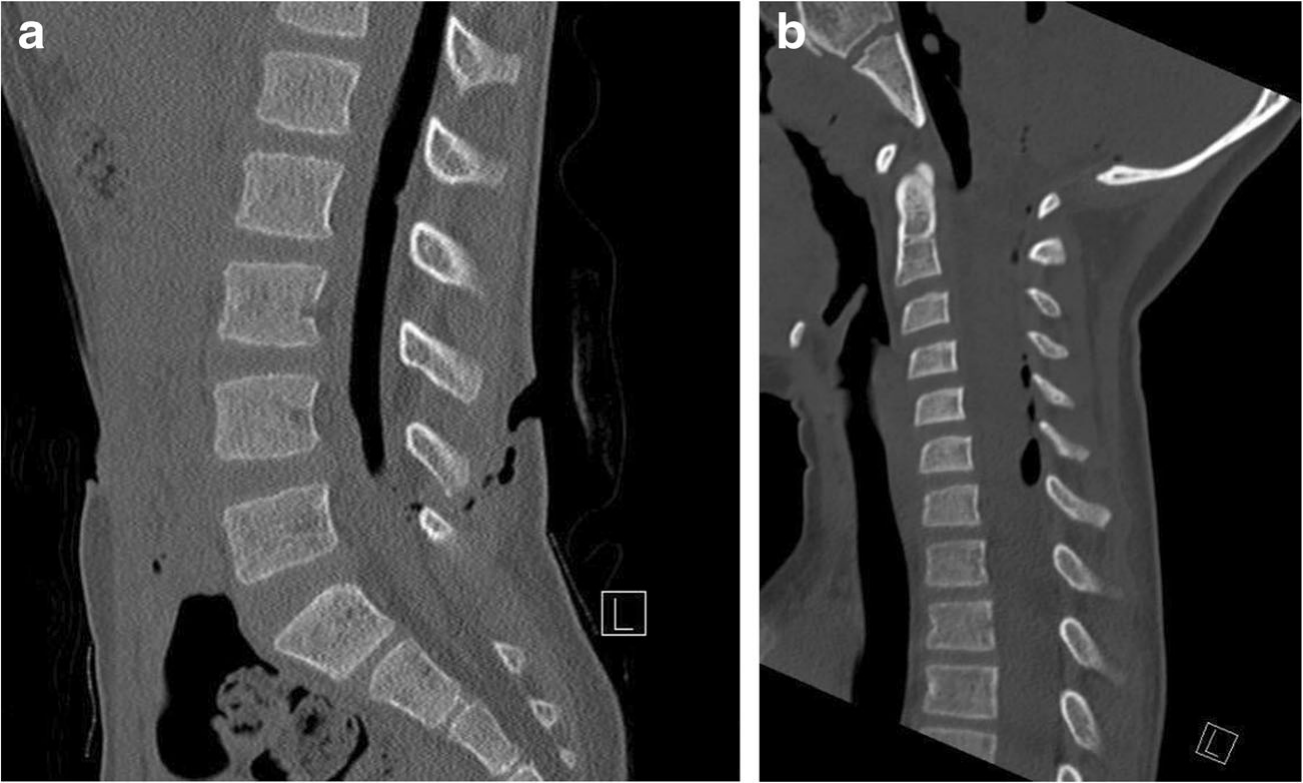
**a** Lumbar CT, sagittal section. **b** Cervical CT, sagittal section

Following the hospital’s trauma protocol, a CT full spine was performed. CT imaging showed a large collection of intraspinal, most probable subdural air, ascending from the low-lumbar wound between the spinous processes of L4 and L5, upwards to the cervical spine and dorsal of the clivus (Fig. 2). Furthermore, a small chip fracture of the caudal part of the spinous process of L4 was visualized (not shown). There were no other spinal fractures.

**Fig. 3:**
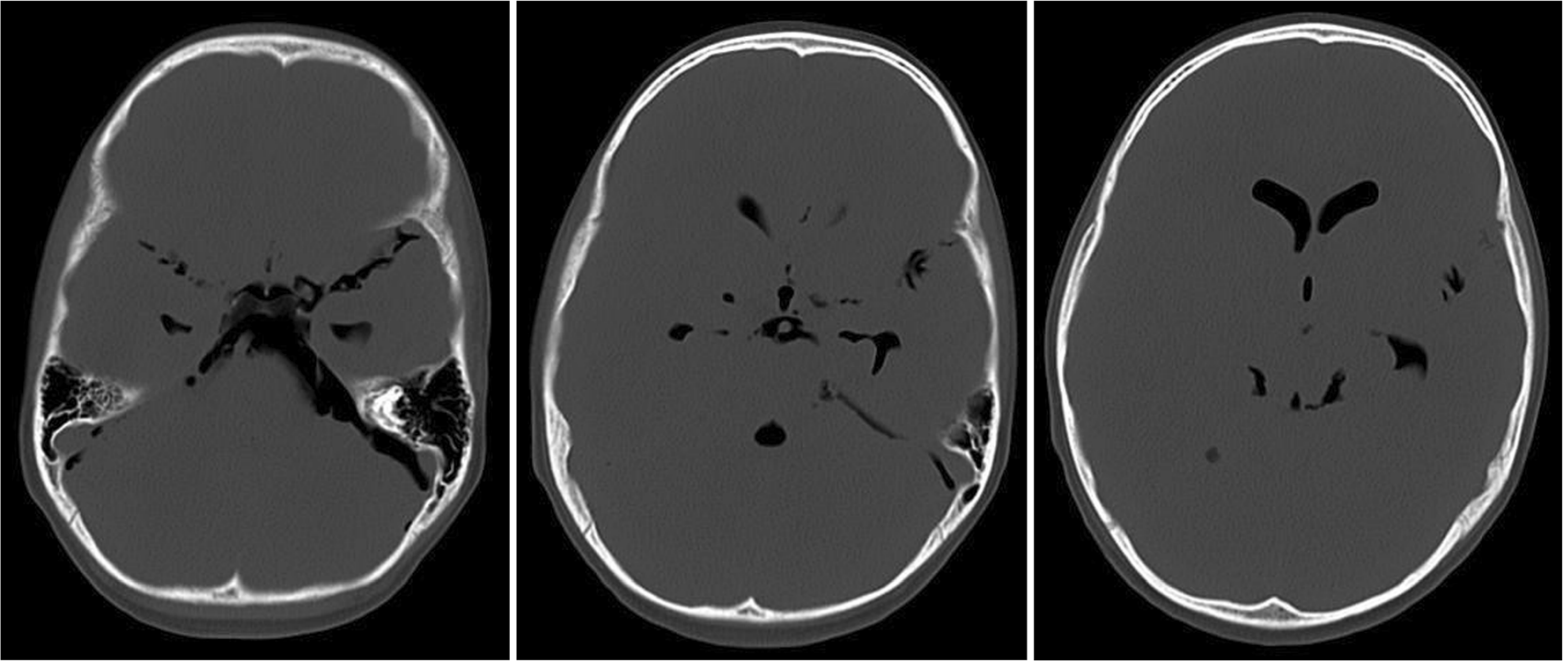
Cerebral CT, axial section, bone setting

Because of the lowered conscious state and the presence of pneumorrhachis, an additional CT scan of the brain was performed. This showed an extensive amount of intracranial air, within all ventricles, prepontine, and subarachnoidally (Fig. 3).

## Diagnosis and treatment

The neurological deterioration resulting in a decreased conscious state and the large collection of intraspinal and intracranial air necessitated us to perform an emergency surgical procedure. An additional decisive argument was the suspicion of persistent leakage of cerebrospinal fluid (CSF) as the cause of the pneumocephalus and as a potential source of meningitis.

**Fig. 4:**
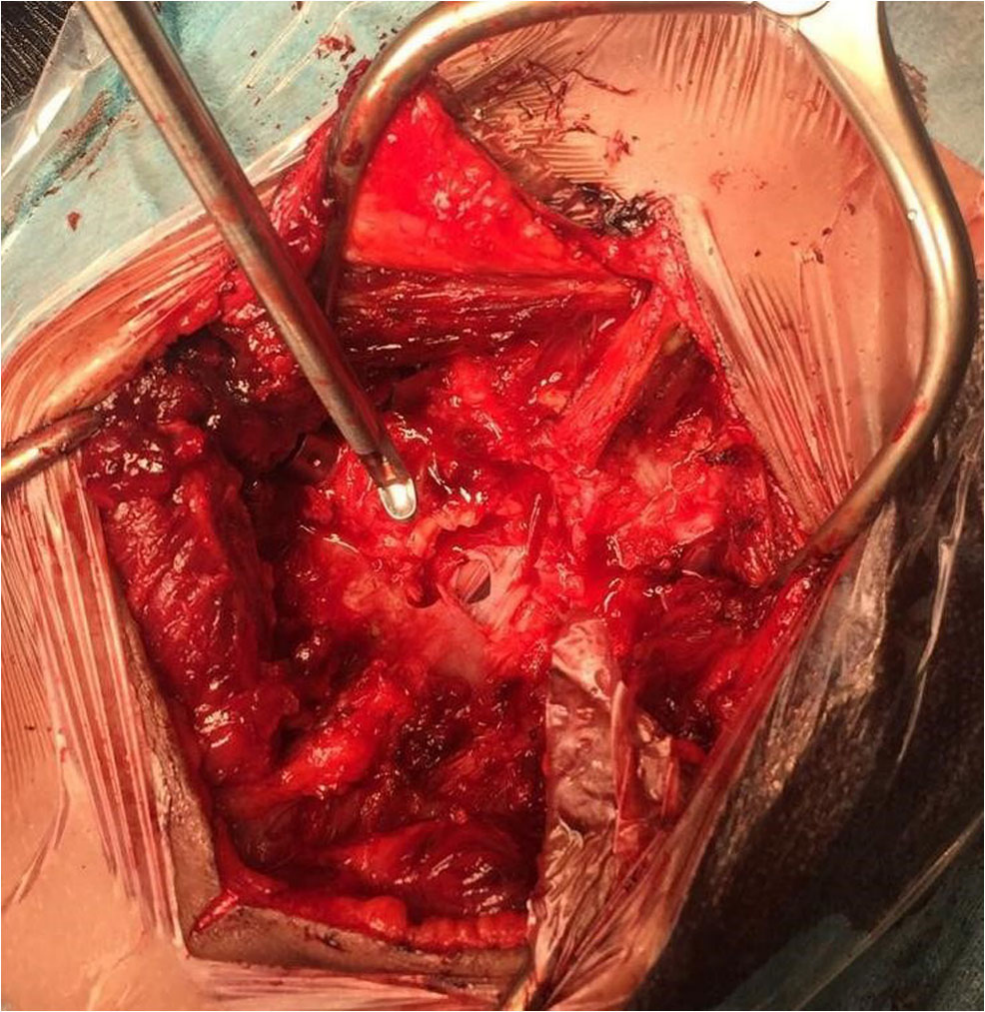
Preoperative view, after laminectomy L4 and flavum decompression, of the dural laceration

The wound was further opened with a Z-plasty. At the level L4–L5, traumatic rupture of the supraspinous, interspinous, and flavum ligaments was found. A L4 laminectomy, followed by flavectomy L3–L4 and L4–L5 was performed. An apparent laceration of the dura was visualized, with an intact filum terminale and cauda equina nerves (Fig. 4). The dural defect was primarily sutured with Vicryl 3-0, followed by sealing with a mixed layer of TachoSil®, TISSEEL®, and Surgicel®.

Afterwards, no more visible CSF leakage was observed. A L4–L5 fusion with pedicle screw instrumentation was carried out because of potential instability, as a result of extensive traumatic ligamentous rupture in combination with surgical bony decompression. Prophylactic cefazolin was administered preoperatively and postoperatively continued for 24 h.

After surgery, the patient woke up alert and adequate. Initially, bed rest in low Fowler’s position was pursued. There was no neurological deficit and the back pain and his preferential head position disappeared. A MRI scan of the brain performed 1 day postoperatively showed an extensive reduction in intracranial air. Spinal x-ray demonstrated a normal lordotic curvature of the spine and correct pedicular screw placement.

Two days postoperatively, the patient developed fever, headache, and meningismus. On high suspicion of meningitis, he was treated with intravenous flucloxacillin and ceftazidime for 2 weeks. A lumbar punction to obtain CSF was unsuccessful, and both urine and blood cultures were negative. During antibiotic treatment, the patient recovered well. Wound healing was uncomplicated. Ten days postoperatively, a CT scan of the brain was performed; almost all intracranial air was resorbed. A couple of days later, the patient was discharged in good clinical condition. Three months postoperatively, the patient was completely recovered. Spinal x-ray showed a normal spinal position and good pedicular screw placement.

## Discussion

Pneumocephalus, also known as intracerebral aerocèle or pneumatocèle, is the presence of intracranial air and can be present intra- or extra-axially [14].

Even though pneumocephalus is frequently encountered on routine imaging after craniotomy in neurosurgical practice, a very limited number of publications about pneumocephalus is available, mostly consisting of case reports, and contains only few epidemiological data [13].

The main cause of pneumocephalus in 75% of patients is neurotrauma, especially with the presence of skull base fractures [2, 7, 14]. Causes in the other 25% are tumors, infections, and iatrogenic causes, the last two after cranial and spinal surgical procedures or local lumbar procedures [5, 8, 14, 19]. In very few case reports, pneumocephalus as a complication of spinal trauma, like thoracic or cervical stab wounds, is described. Here, patients were treated conservatively [10, 11, 17]. However, a rapid decrease in consciousness after lumbar trauma, with extensive amounts of intraspinal and intracranial air demanding for emergency surgery, has not been reported.

Pneumocephalus is mostly asymptomatic. But, it may cause headache, confusion, nausea, vomiting, seizures, dizziness, and/or focal neurological symptoms, like hemiparesis and/ or cranial nerve palsy [7, 14, 16].

In clinical practice, it is of utmost importance to differentiate between a pneumocephalus and a tension pneumocephalus [14, 16], since a tension pneumocephalus should be considered as a neurosurgical emergency and will require immediate action. An untreated tension pneumocephalus may lead to cerebral herniation with coma or death as a result [2, 15, 16].

The pathophysiology of pneumocephalus can be explained by two mechanisms: “the inverted soda bottle mechanism” and “the ball valve mechanism.” “The inverted soda bottle mechanism” states that continuous leakage of CSF results in a negative intracranial pressure in the subarachnoid space. As a result, air can be drawn in through an existing dural defect. The air will ascend and replace the CSF that leaked out, until the pressure gradient is stable [3, 7, 16]. In “the ball valve mechanism,” the mechanism of action in tension pneumocephalus, a pressure gradient arises and causes a large collection of intracranial air [2, 3]. This pressure gradient develops when extracranial pressure exceeds intracranial pressure, and when a dural injury is present. Pressure-increasing moments, like coughing, induce a sudden pressure increase in the paranasal cavities, which pulls air intracranially through the dural defect [3, 16, 19]. The intracranial tissue then blocks the dural entrance, preventing the air from flowing back [15, 19].

Clinically, it is difficult to distinguish between the two types of pneumocephalus. The gold standard for diagnosis is CT, with the high sensitivity and high specificity able to detect even small amounts of air [2, 14]. The most important CT findings are the “peaking sign,” “Mount Fuji sign,” and the “air bubble sign” [2, 14]. A “peaking sign,” in which there is bilateral compression without separation of the frontal lobes, is often seen in the initial stage of upcoming pneumocephalus and less indication for a tension pneumocephalus [2]. The second stage, “Mount Fuji sign,” is caused by the presence of subdural air around and between both frontal lobes, creating an image similar to Mount Fuji’s silhouette [4]. Separation of the tips of the frontal lobes reveals the presence of subdural air exceeding the tension of CSF, and more characteristic for tension pneumocephalus [4]. Finally, the presence of multiple small air bubbles scattered through several cisterns and the arachnoid space is called “air bubble sign” [4]. However not pathognomonic, both “Mount Fuji sign” and “air bubble sign” may indicate the development of tension pneumocephalus [2, 4, 14]. Given the benign nature of pneumocephalus, treatment is usually conservative and consists mainly of pursuing strict bed rest in a low Fowler’s position with a 30° elevation of the head of the patient in bed, to promote good cerebral oxygenation by better chest expansion compared to the supine position with or without flexion in the legs [1, 6]. Further conservative treatment involves administration of antipyretic pain medication to prevent hyperthermia, avoidance of the Valsalva maneuver (e.g., coughing), and excessive physical activities. These recommendations form an effective approach with a reduction of intracranial air in 85% of patients, within 2 or 3 weeks [8, 12].

Another treatment option is hyperbaric oxygenation (HBO2) therapy [1]. Therapy with HBO2 promotes a reduction of the volume of intracranial air in patients with symptomatic pneumocephalus by an accelerated clearance of nitrogen, compared to treatment with normobaric hyperoxygenation. Furthermore, HBO2 therapy seems to reduce the incidence of meningitis and hospital stay and shows no adverse side effects [9, 18]. In all cases, it is important to monitor the decline of intracranial air over time with CT [14].

In contrast, suspicion of tension pneumocephalus implies an invasive approach. If possible, the cause of pneumocephalus should be treated, e.g., by closing the underlying dura defect. An urgent burr hole or decompressive craniotomy to immediately decrease the intracranial pressure by aspiration of air should be strongly recommended in case of rapid neurological decline [14].

In the unique case outlined above, a CT scan of the brain did not show the typical characteristics of tension pneumocephalus, which is probably the result of the trauma mechanism. Since the dural defect was localized in the lumbar region, the local pressure gradient was very large in the upright position, estimated on at least 40 cmH2O based on the patient’s length. The ascended intracranial air could impossibly escape through the lower situated dural defect. Therefore, we hypothesize an “enhanced inverted soda bottle mechanism” to be responsible.

## Conclusion

The presented unique pediatric case illustrates the occurrence of life-threatening pneumocephalus after a penetrating lumbar spinal injury associated with a local dural defect. All patients with rapid neurological deterioration after spinal trauma and suspicion of CSF leakage should be treated as having the clinical diagnosis of a tension pneumocephalus. This is especially important for pediatric patients. These patients need emergency surgery to close the dural defect, which will result in a quick and complete recovery of the patient.
